# Second and third look laparoscopy in pT4 colon cancer patients for early detection of peritoneal metastases; the COLOPEC 2 randomized multicentre trial

**DOI:** 10.1186/s12885-019-5408-8

**Published:** 2019-03-21

**Authors:** Vivian P. Bastiaenen, Charlotte E. L. Klaver, Niels F. M. Kok, Johannes H. W. de Wilt, Ignace H. J. T. de Hingh, Arend G. J. Aalbers, Djamila Boerma, Andre J. A. Bremers, Jacobus W. A. Burger, Eino B. van Duyn, Pauline Evers, Wilhelmina M. U. van Grevenstein, Patrick H. J. Hemmer, Eva V. E. Madsen, Petur Snaebjornsson, Jurriaan B. Tuynman, Marinus J. Wiezer, Marcel G. W. Dijkgraaf, Jarmila D. W. van der Bilt, Pieter J. Tanis

**Affiliations:** 1 Department of Surgery, Amsterdam UMC, University of Amsterdam, Cancer Center Amsterdam, PO Box 22660, 1105 AZ Amsterdam, the Netherlands; 2 Department of Surgery, the Netherlands Cancer Institute, Amsterdam, the Netherlands; 3 Department of Surgery, Radboud University Medical Centre, Nijmegen, the Netherlands; 4 Department of Surgery, Catharina Hospital, Eindhoven, the Netherlands; 5 Department of Surgery, St. Antonius Hospital, Nieuwegein, the Netherlands; 6 Department of Surgery, Medisch Spectrum Twente, Enschede, the Netherlands; 7 Dutch Federation of Cancer Patient Organizations (NFK), Utrecht, the Netherlands; 8 Department of Surgery, University Medical Centre Utrecht, Utrecht University, Utrecht, the Netherlands; 9 Department of Surgery, University Medical Centre Groningen, University of Groningen, Groningen, the Netherlands; 10 Department of Surgery, Erasmus MC, Rotterdam, the Netherlands; 11 Department of Pathology, the Netherlands Cancer Institute, Amsterdam, the Netherlands; 12 Department of Surgery, Amsterdam UMC, Vrije Universiteit Amsterdam, Cancer Center Amsterdam, Amsterdam, the Netherlands; 13 Department of Clinical Epidemiology, Biostatistics and Bioinformatics, Amsterdam UMC, University of Amsterdam, Amsterdam, the Netherlands; 14 Department of Surgery, Flevo hospital, Almere, the Netherlands

**Keywords:** T4 colon cancer, Peritoneal metastases, Early detection, Diagnostic laparoscopy, Second/third look surgery

## Abstract

**Background:**

Approximately 20–30% of patients with pT4 colon cancer develop metachronous peritoneal metastases (PM). Due to restricted accuracy of imaging modalities and absence of early symptoms, PM are often detected at a stage in which only a quarter of patients are eligible for curative intent treatment. Preliminary findings of the COLOPEC trial (NCT02231086) revealed that PM were already detected during surgical re-exploration within two months after primary resection in 9% of patients with pT4 colon cancer. Therefore, second look diagnostic laparoscopy (DLS) to detect PM at a subclinical stage may be considered an essential component of early follow-up in these patients, although this needs confirmation in a larger patient cohort. Furthermore, a third look DLS after a negative second look DLS might be beneficial for detection of PM occurring at a later stage.

**Methods:**

The aim of this study is to determine the yield of second look DLS and added value of third look DLS after negative second look DLS in detecting occult PM in pT4N0-2 M0 colon cancer patients after completion of primary treatment. Patients will undergo an abdominal CT at 6 months postoperative, followed by a second look DLS within 1 month if no PM or other metastases not amenable for local treatment are detected. Patients without PM will subsequently be randomized between routine follow-up including 18 months abdominal CT, or an experimental arm with a third look DLS provided that PM or incurable metastases are absent at the 18 months abdominal CT. Primary endpoint is the proportion of PM detected after a negative second look DLS and will be determined at 20 months postoperative.

**Discussion:**

Second look DLS is supposed to result in 10% occult PM, and third look DLS after negative second look DLS is expected to detect an additional 10% of PM compared to routine follow-up alone in patients with pT4 colon cancer. Detection of PM at an early stage will likely increase the proportion of patients eligible for curative intent treatment and subsequently improve survival, given the uniformly reported direct association between the extent of peritoneal disease and survival.

**Trial registration:**

ClinicalTrials.gov: NCT03413254, January 2018.

**Electronic supplementary material:**

The online version of this article (10.1186/s12885-019-5408-8) contains supplementary material, which is available to authorized users.

## Background

Colorectal cancer (CRC) is the third most common cancer worldwide, with an estimated incidence of over 1.8 million in 2018 [1]. A common site of recurrence in patients with CRC is the peritoneum, which is the sole site of recurrence in up to 25% [2]. Peritoneal dissemination of colorectal origin largely depends on clinical stage and histological subtype and was formerly considered a terminal condition with dismal prognosis. Median survival is only about 5 months if untreated and ranges between 5.2 and 12.6 months if treated with 5-fluorouracil and leucovorin-based systemic chemotherapy [3–5]. Modern systemic therapy regimens have significantly improved patient outcomes. Patients with isolated, resectable peritoneal metastases (PM) have median survival of up to 24 months with oxaliplatin/irinotecan-containing combinations with or without biological agents [6–9]. However, systemic treatment results in lower survival benefit in PM as compared with non-peritoneal metastases, and long-term survival remains limited with 5-year survival probability of only 13% [6, 9, 10].

Currently, the only curative option for PM is cytoreductive surgery (CRS) followed by hyperthermic intraperitoneal chemotherapy (HIPEC). The purpose of this multimodal approach is to surgically remove all visible peritoneal tumour deposits and to eradicate microscopic residual disease with intraperitoneal administration of heated chemotherapy [11]. Previous studies have shown that CRS/HIPEC improves survival in comparison with systemic chemotherapy alone [3, 6, 12–14]. In a systematic review including nineteen cohort studies and thirteen comparative studies published between 2010 and 2015, the weighted median overall survival after treatment with CRS/HIPEC was 31.6 months and the 5-year survival rate ranged between 22 and 51% [8].

The efficacy of CRS/HIPEC highly depends on the extent of peritoneal involvement, which is often assessed with the peritoneal cancer index (PCI) as proposed by Jacquet and Sugarbaker [3, 15–22]. Patients with a low PCI (1–5) have reported median survival of up to 81 months and 5-year survival rates exceeding 70%, whereas only about 10% of patients with extensive PM (PCI ≥16) are alive at 5 years postoperative [23, 24]. Besides a survival benefit, limited extent of peritoneal disease is also correlated with lower postoperative morbidity following CRS [15, 17, 19, 25]. Furthermore, in patients with a low PCI, less extensive surgery is required to achieve completeness of cytoreduction, which is another important prognostic factor for survival [3, 6, 15–19, 25–27].

The availability of an effective therapy, and the fact that CRS/HIPEC yields better survival rates and lower postoperative morbidity when the extent of peritoneal disease is more limited, underline the importance of detection and treatment of PM in its initial stages. However, early PM cannot be detected with clinical or radiological methods due to the absence of symptoms and restricted accuracy of imaging modalities. Sensitivity of CT for the detection of PM ranges from 60 to 79%, but drops below 30% in case of peritoneal lesions smaller than 5 mm [28]. As a result, PM are often detected at a stage in which only about 20–25% of patients can be treated with CRS/HIPEC. Therefore, new diagnostic strategies are urgently required to detect PM at a clinically occult stage, which will probably result in a higher percentage of patients eligible for intentional curative treatment. This is supposed to translate into better survival, considering the direct association between PCI and survival.

Currently, the only way to diagnose minimal PM is by re-exploration of the abdominal cavity during second look surgery. The concept of second look surgery was first described by Wangensteen in 1948 and is based on the systematic use of planned reoperation in asymptomatic patients who are theoretically at risk for developing recurrent or metastatic disease, despite initial curative resection of the primary tumour [29]. Since it is an invasive and costly procedure, second look surgery should only be proposed to selected patients at high-risk of developing PM. An advanced stage of colon cancer has been shown to be an important risk factor for the development of PM [30, 31]. After curative resection of a pT4 primary tumour, the risk of developing metachronous PM is approximately 30% [32].

In 2017, our study group completed the accrual of 204 patients in the COLOPEC randomized controlled trial (NCT02231086), which investigated the effect of adjuvant HIPEC preceding routine adjuvant systemic therapy on the outgrowth of PM after resection of pT4 or perforated colon cancer [33]. In most patients, adjuvant HIPEC was applied as a staged procedure 5–8 weeks after resection of the primary tumour. As part of the trial, routine diagnostic laparoscopy (DLS) at 18 months was performed in both arms to determine the effectiveness of adjuvant HIPEC based on the 18 months peritoneal metastases free survival (PMFS, primary endpoint). Results of the primary endpoint are expected at the beginning of 2019. However, PM were already detected during surgical exploration, which was part of the planned adjuvant HIPEC procedure, in 9% of patients (preliminary unpublished data). Based on these findings and literature, a second look DLS to detect PM when the disease is still potentially curable by CRS/HIPEC may be considered an essential component of early follow-up of pT4 colon cancer patients. This finding needs confirmation in a larger patient cohort before implementing this into routine practice. Some patients develop metachronous PM at a longer interval (> 12 months) from primary resection, and those patients will be missed by a second look DLS [34]. For this reason, there might be benefit of a third look DLS after a negative second look DLS to detect occult metachronous PM later on. In the COLOPEC trial, a quarter of detected PM among both arms of the study were found at 18 months DLS after negative CT (preliminary unpublished data). These findings will be further explored in the COLOPEC 2 trial.

## Methods/design

This study protocol is written in accordance with the SPIRIT guidelines [35, 36]. The SPIRIT Checklist is provided in Additional file 1.

### Objectives

The primary objectives of this study are to determine the yield of second look DLS and the added value of a third look DLS after a negative second look DLS in patients who underwent resection of pT4a,bN0-2 M0 colon cancer for the detection of clinically occult PM which are amenable for curative intent treatment. Main secondary objectives are to determine the 5-year peritoneal metastases free, disease-free and overall survival.

### Design

**Fig. 1:**
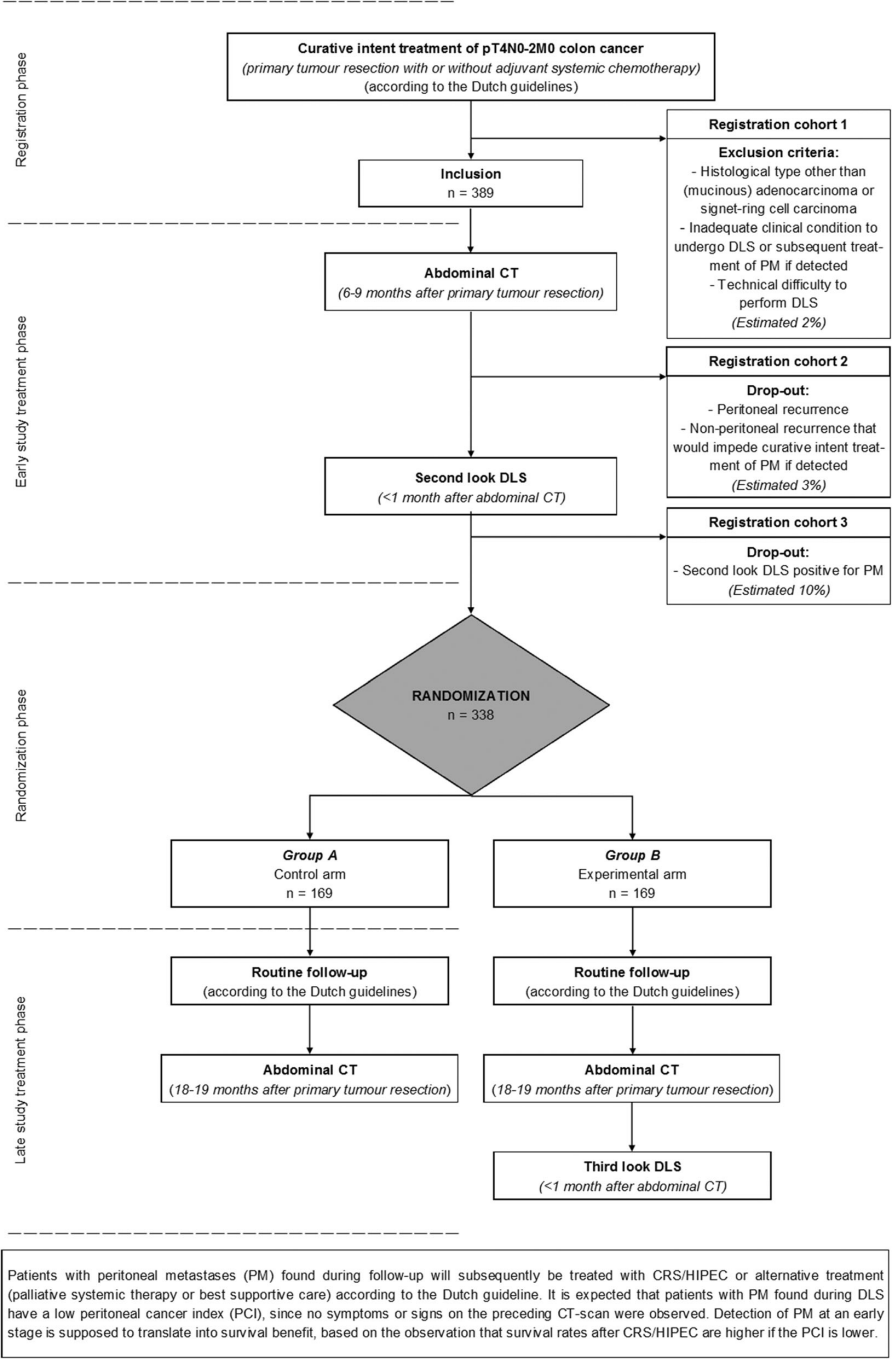
Flowchart. CT, computed tomography; DLS, diagnostic laparoscopy; PM, peritoneal metastases

This is a prospective multicentre randomized controlled trial of the Dutch Peritoneal Oncology Group (DPOG) and Dutch Colorectal Cancer Group (DCCG) with a two-armed parallel design and 1:1 allocation ratio. This study started in February 2018 and will be performed in approximately 30 Dutch hospitals, both HIPEC centres and non-HIPEC centres. Eligible patients will undergo an abdominal CT at six months postoperative. For patients who still have to finish adjuvant systemic chemotherapy, a maximum delay of three months will be allowed for this CT scan. If the CT scan is negative for peritoneal recurrence or other metastases that are not curable, a second look DLS will be performed within one month from this CT scan. Patients without PM during the second look DLS will subsequently be randomized to routine follow-up including an abdominal CT at 18 months followed by third look DLS in the experimental arm, or routine follow-up including an abdominal CT at 18 months alone in the control arm (Fig. 1). A maximum delay of one month is allowed for the 18 months CT scan. The third look DLS in the experimental arm will not be performed in patients with:

- PM found during routine follow-up or on CT-imaging;
- Non-peritoneal recurrence that would impede curative intent treatment of PM if detected, according to the local multidisciplinary team.

Patients in whom PM are found at any time during follow-up will be treated by CRS/HIPEC or alternative treatment (palliative systemic therapy or best supportive care) according to the Dutch guideline and patient preferences [37].

### Study population

Patients who underwent intentional curative resection of pT4a,bN0-2 M0 colon cancer or rectosigmoid cancer above the peritoneal reflection, with or without adjuvant systemic chemotherapy, will be considered for inclusion.

In order to be eligible to participate in this study, a subject must meet all of the following criteria:

- Age between 18 and 80 years;
- Written informed consent.

A potential subject who meets any of the following criteria will be excluded from participation in this study:

- Histological types other than adenocarcinoma, mucinous adenocarcinoma or signet-ring cell carcinoma;
- Clinical condition that does not allow for second look surgery or subsequent treatment of PM if detected;
- Second look surgery thought not to be technically possible (i.e. because of extensive abdominal surgery / re-interventions).

### Enrolment

Eligible patients will be approached for entry into the study at the first outpatient visit after resection of the primary tumour. The rationale for the study will be explained to the patient. A patient information sheet is provided and patients will be given the opportunity to ask questions. After a sufficient reflection period, patients are asked to sign the consent form. Written informed consent is taken by surgeons, surgical registrars or trained research nurses prior to any study procedures. When consent has been obtained, the original form is kept in the study file and a copy is given to the patient. The study coordinator will register all included patients in a registration module built with ALEA (FormsVision). Every registered patient will be assigned a sequential subject number consisting of three digits. A notification of the registration will be sent to the local investigator of the site where the patient was included. A log of the assigned subject numbers will be maintained by each site.

### Standard care between inclusion and randomization

Blood samples to determine carcinoembryonic antigen (CEA) levels are collected at 3, 6 and 9 months postoperative. At 6–9 months after resection of the primary tumour with or without adjuvant systemic chemotherapy, CT imaging of the abdomen is performed in all included patients who are in an adequate clinical condition to undergo DLS. All patients without signs of PM or non-peritoneal recurrence that would impede curative treatment of PM if detected on CT will undergo a second look DLS. The surgical procedure is described below.

### Randomization

Randomization will take place after the second look DLS. Patients without PM during second look DLS will be randomized in a 1:1 ratio between routine follow-up including an abdominal CT at 18 months followed by third look DLS (experimental arm) and routine follow-up including an abdominal CT at 18 months alone (control arm). Randomization with permuted blocks of random sizes will be performed by the study coordinator using the registration module built with ALEA (FormsVision). The block sizes will not be disclosed to ensure concealment. Stratification factors will be the surgical approach of the primary tumour resection (laparoscopy or open), pathological nodal stage (N0 or N1–2), and adjuvant systemic chemotherapy (yes or no, yes: at least four cycles of capecitabine and oxaliplatin (CAPOX) or six cycles of 5-fluorouracil, leucovorin and oxaliplatin (FOLFOX), allowing dose reduction and omission of oxaliplatin, no: all other cases) [30, 31]. In order to prevent caretakers from being influenced by the assigned follow-up strategy, the randomization outcome will remain unknown to participants and everyone involved in the patient’s care until the 18 months CT scan of the abdomen is reported by the radiologist. Once the radiology report of the abdominal CT is available, a notification with the assigned allocation is sent to the local investigator of the site where the patient was included.

### Standard care of the control arm

Follow-up following negative second look DLS will be performed routinely according to the Dutch guideline [37]. Patients visit the outpatient clinic twice a year during the first two to three years and annually thereafter, until five years postoperative. CEA-levels are determined at 12, 15, 18, 21, 24, 30, 36, 48 and 60 months after primary resection. Imaging of the liver using ultrasound or CT is performed at 12, 24, 36, 48 and 60 months postoperative. At 18 months postoperative, an abdominal CT is part of the study protocol, but ultrasound of the liver is allowed for other time intervals during follow-up. Colonoscopy is performed at 12 and 48 months postoperative. The follow-up schedule is displayed in Table 1.

**Table 1:** Routine follow-up schedule

TIMEPOINT *months after primary resection*	3	6	9	12	15	18	21	24	30	36	48	60
CEA	X	X	X	X	X	X	X	X	X	X	X	X
Abdominal CT		X	X^a^			X						
Ultrasound / CT liver				X				X		X	X	X
Colonoscopy				X							X	
Diagnostic laparoscopy		X	X^a^			X^b^						

*CEA* carcinoembryonic antigen, *CT* computed tomography

^a^In case of delay related to adjuvant systemic chemotherapy

^b^ According to randomization

### Standard care of the experimental arm

Follow-up in the experimental arm is similar to the control arm except for a third look DLS following the abdominal CT at 18 months. Third look DLS is not performed in patients with evidence of disease that is not curable, or in those already diagnosed with PM in the preceding period.

#### Second and third look DLS

Surgeons experienced in performing DLS for the detection of PM will proctor the procedure in the participating non-HIPEC centres. After proctoring, surgeons in the participating non-HIPEC centres are asked to provide videos from their first three individually performed cases in order to monitor their skills. The patient is placed in French position on a bean bag under general anesthesia. Open introduction of a 10–12 mm trocart is performed outside areas of expected adhesions (i.e. Palmer’s point) and a CO2 pneumoperitoneum is created. Additional trocars of 5 mm are placed under direct vision to allow for adequate adhesiolysis and inspection. All regions of the abdominal cavity, including the local resection site, are systematically and thoroughly inspected. Adhesiolysis is performed if necessary, and extent of adhesiolysis should be determined based on an optimal balance between improved visualization and risk of (bowel) damage. Conversion to laparotomy if there is inadequate exposure during DLS is prohibited, while this is considered to be too invasive for diagnostic purposes in this trial setting according to the members of the research committee of the DPOG. If adequate exploration of the abdomen appears not to be possible by laparoscopy, the procedure has to be stopped. It is only allowed to convert to laparotomy in case of an intra-operative complication that requires intervention by an open approach. The PCI score is used to assess the extent of peritoneal disease if present. Samples from lesions suspicious for PM are taken and sent to the pathology lab. After inspection of the entire abdominal cavity, trocars are removed and port sites are closed in standard fashion. Patients will be treated in an outpatient surgery setting and will be discharged from the hospital if they meet all discharge criteria.

DLS is associated with a low risk of wound infection of the trocar sites, bleeding from the abdominal wall or biopsy sites, and bowel injury related to adhesiolysis. With an expected number needed to diagnose clinically occult PM of 10, DLS related morbidity will probably not outweigh the potential survival benefit related to higher proportions of curative intent treatment compared to detection at a clinically apparent stage.

### Outcomes

#### Primary study endpoint

The primary endpoint of the study is the proportion of PM detected after a negative second look DLS. The primary endpoint will be determined at 20 months.

#### Secondary study endpoints

Secondary endpoints of the COLOPEC II trial are:

- Incidence of PM at second look DLS and at 20 months after curative resection of the primary tumour, depending on pT4 subdivision, pathological nodal stage, histology and receipt of adjuvant chemotherapy;
- Proportion of PM at 20 months after curative resection of the primary tumour that was detected with routine follow-up including abdominal CT at 18 months in both arms and with third look DLS in the experimental arm;
- Proportion of detected PM eligible for CRS + HIPEC at different follow-up intervals;
- Clinical manifestation of PM within 6 months of the second look DLS;
- Sensitivity, specificity, positive predictive value (PPV) and negative predictive value (NPV) of CT imaging to detect PM;
- Thirty-day morbidity related to second/third look DLS;
- Extent of adhesions assessed with the Zühlke score and need for adhesiolysis at second/third look DLS [38];
- Peritoneal recurrence free survival rate at 5 years;
  - In patients with a negative second look DLS;
  - In patients with a negative 18 months abdominal CT who did or did not subsequently undergo third look DLS.
- Disease-free survival rate at 5 years;
  - In patients with a negative second look DLS;
  - In patients with a negative 18 months abdominal CT who did or did not subsequently undergo third look DLS.
- Overall survival rate at 5 years;
  - In patients with a positive second look DLS;
  - In patients with a negative second look DLS;
  - In patients with a negative CT-abdomen at 18 months who did or did not subsequently undergo third look DLS.
- Quality of life at 1 year and 2 years (CRC-29; EQ-5D-5 L).

### Sample size calculation

It is expected that 87% of included patients will have negative second look DLS (Fig. 1). These patients will be randomized. It is expected that about 5% will be diagnosed with irresectable metastases located outside the peritoneum between 6 and 18 months postoperative, and another 2% will present themselves with clinically manifest PM before the 18 months CT. The abdominal CT at 18 months postoperative will presumably identify another 3% true positives (3.06% testing positive; positive predictive value of CT: 0.98). Hence, it is expected that PM will be detected in about 5% of patients in the control arm at 20 months. Assuming a negative predictive value of the 18 months CT for PM of 0.89 and assuming that the third look DLS followed by evaluation by the pathologist if biopsies were taken will identify all CT false negatives, it is expected that, overall, PM will be detected in approximately 15% of patients in the experimental arm. This results in an absolute difference of 10%, which is considered to be clinically relevant. If 320 patients (160 per group) with negative second look DLS are randomized, a Fisher’s exact test for two independent proportions with a 0.025 one-sided significance level will have 80% power to detect an absolute difference of 10% in actual PM at 20 months (about 5% under routine follow-up and approximately 15% under third look surgery at 18–20 months). Because of an expected dropout rate of 5%, 338 subjects will be randomized to ensure that a minimum sample size of 320 patients is obtained. In order to achieve this number of patients, 338/0.87, or 389 patients should initially be included. The calculation was based on PASS 2005 software.

### Recruitment

The Dutch ColoRectal Audit (DCRA, former DSCA) revealed that in 2011 approximately 10.500 patients were diagnosed with colon cancer, of which 14% presented with a pT4 stage colon carcinoma. Despite the introduction of bowel cancer screening, this percentage has not decreased, probably due to better histological diagnosis of peritoneal penetration related to more awareness of its clinical consequences. In 2016, more than 1600 patients (15%) were diagnosed with a pT4 stage colon tumour. Based on an estimated drop-out of 15% due to age older than 80 years, the estimated number of eligible patients for the COLOPEC 2 trial is about 1.350 each year. During the COLOPEC trial, a wide network of surgeons throughout the Netherlands was built and patient accrual went faster than expected. The COLOPEC 2 trial will be performed in the same centres as in the COLOPEC trial, so the participating centres are familiar with performing randomized controlled trials and the surgeons are aware of the risk of pT4 colon cancer patients to develop PM. Therefore, it is expected that patient accrual can be completed in three years.

### Data collection and data management

Data collection and data management will be performed by the Netherlands Comprehensive Cancer Organization (IKNL). Their experience with continuous data collection based on high quality electronic case report forms (eCRFs) guarantees complete and timely recording, handling and storage of data and documents. All local and central data managers are registered and the electronic database (TRIAS) is ISO certified. Data will be documented in line with ‘Good Clinical Practice (GCP)’ and Dutch legal requirements. Major violations of the protocol will be recorded.

In all participating hospitals, one surgeon acts as local investigator who is primarily responsible for practical execution of the trial in compliance with the study protocol. The local investigators will be responsible for accuracy and completeness of data. Data will be registered in the patient file by the treating physician. In every hospital, a local data manager of IKNL is responsible for entering the data in the electronic database. After inclusion, baseline characteristics including patient and tumour characteristics will be retrieved from patients’ charts. During the DLS, information about the surgical procedure will be collected by the surgeon on a paper case record form. During the 30-day postoperative period, adverse events will be reviewed and documented. During routine outpatient clinic visits for oncological follow-up at 3, 6, 12, 18, 24, 36, 48 and 60 months, disease recurrence will be checked. Quality of life assessments will be done with the CR-29 questionnaire and the EQ-5D-5 L health status questionnaire. In case a subject has withdrawn, the reason for withdrawal will be documented and no further study assessments will be performed. Subjects whom have been withdrawn from the study but are still willing to participate in the routine follow-up will be followed according to the specifications of the patient. The schedule for enrolment, interventions and assessments is summarized in Table 2.

**Table 2:**
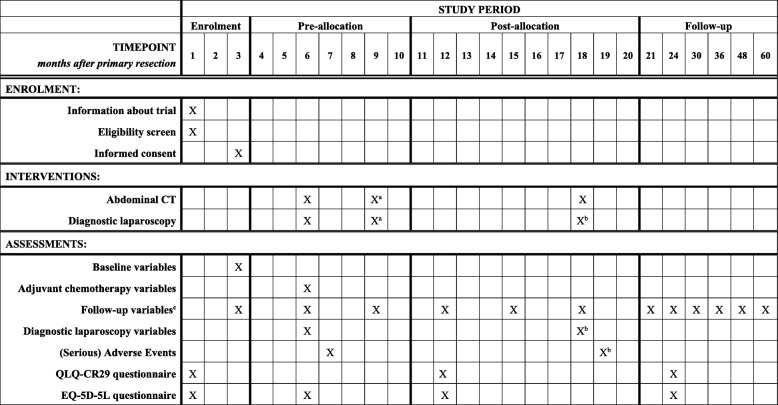
Schedule of enrolment, interventions and assessments

*CT* computed tomography

^a^ In case of delay related to adjuvant systemic chemotherapy

^b^ According to randomization

^c^ Including peritoneal metastases assessment

The central data manager of IKNL develops the eCRFs including range checks for data values, adds participating hospitals to the database, tests the database, and informs the local datamanagers about how to use the database. The central datamanager assures the progress of data collection, will maintain quality of documentation by local data managers in the eCRF, and clarifies mistakes where necessary. In case of uncertainties or questions in the eCRF, additional queries for the local data managers may be formulated by the central datamanager.

### Data analysis

All analyses described below will be performed in the newest SPSS-version at the time of analysis. A *p*-value below 0.05 will indicate statistical significance in case of formal testing of differences between diagnostic strategies. No correction for multiple testing will be applied.

#### Primary study endpoint

Proportions of patients with detected PM following a negative second look DLS will be reported per diagnostic strategy, including the 95% confidence intervals. The difference in proportions between both diagnostic strategies will be tested with the Fisher’s exact test. Additional analyses by Cochran-Mantel-Haenszel testing will be performed to account for the stratification variables. Hardly any missing primary outcome data are expected, e.g. the nationwide study design allows for study continuation by patients if they happen to move to another region.

#### Secondary study endpoints

Incidences of PM at different time points, depending on pT4 subdivision, pathological nodal stage, histological type and receipt of adjuvant chemotherapy will be descriptively reported per diagnostic strategy as rates per 100 patients, along with their 95% confidence intervals. Similar descriptive analyses will be applied to the (i) proportion of PM at 20 months after curative resection of the primary tumour that was detected with routine follow-up including abdominal CT at 18 months in both arms and with third look DLS in the experimental arm, (ii) the proportion of detected PM eligible for curative intent CRS + HIPEC at different follow-up intervals; (iii) clinical manifestation of PM within 6 months of second look DLS. No formal testing of differences between diagnostic strategies will be applied to these incidence rates and proportions, but the derived rate ratios will be reported along with the 95% confidence intervals to explore how both diagnostic strategies perform in various subgroups of patients.

Using pathology results or clinical progression as the reference standard, the sensitivity, specificity, PPV and NPV of CT for the diagnosis of PM will be calculated and reported as proportions along with their 95% confidence interval. The 30-day morbidity related to second/third look DLS will be tabulated as counts by type of morbidity, allowing patients to contribute to different types of morbidity. In addition, the distribution of the number of morbidities per patient will be reported. The extent of adhesiolysis required at second/third look DLS will be reported in tabular format as counts for the second as well as third look DLS.

Kaplan-Meier survival analyses for patients with negative second look DLS will be applied to describe the time till disease recurrence and time till death for each diagnostic strategy, censoring patients without disease recurrence, respectively still alive at the end of follow-up (study closure or day of lost-to-follow-up). Differences between diagnostic strategies in time till disease recurrence or time till death will be assessed with the log-rank test. Similar Kaplan-Meier survival analyses will be used for patients with a negative abdominal CT at 18 months as well as patients with a positive second look DLS to describe the time till death.

Generalized linear mixed modelling will be applied to assess differences in quality of life between both diagnostic strategies over time. Mixed models are considerably robust against the presence of missing data.

### Monitoring

No data and safety monitoring board (DSMB) will be assigned, since patients are subjected to an intervention with a low postoperative morbidity that is already being performed in routine clinical practice. No interim analyses will be performed.

### Auditing

Independent monitoring of the study progress and study quality is performed by a qualified monitor of IKNL. The monitor plan is based on the judgement of the IRB that study participation is of moderate risk. Monitoring will be done by exploring the electronic trial database and performing site visits. Each participating site will be visited at least once, with repeat visits to sites where performance is a concern. The quality assessment will focus on the safety, wellbeing and rights of the patients, the quality of the documented data in the eCRF and their traceability to source documents and the completeness of the regulatory binder. Furthermore, the trial monitor checks if the study is executed according to the study protocol, GCP and the declaration of Helsinki. After each monitor visit, the trial monitor reports feedback to the project leader, study coordinator and local investigator.

### Adverse events

All adverse events (AEs) and serious adverse events (SAEs) reported spontaneously by the subject or observed by the investigator or his staff in the first 30 days after second/third look DLS will be recorded. The study coordinator will report all SAEs to the accredited Institutional Review Board (IRB) that approved the study protocol. The clinical course of each AE will be followed until resolution, stabilization or until it has been determined that study treatment or participation is not the cause. SAE’s, which are still ongoing at the end of the study period, must be followed up to determine the final outcome.

### Ethics

The IRB of the Amsterdam UMC, location AMC, has approved the study protocol (MEC 2017_134, NL61507.018.17). All amendments will require formal approval of the IRB prior to implementation. This study will be conducted according to the principles of the Declaration of Helsinki (Fortaleza, October 2013) and in accordance with the Medical Research Involving Human Subjects Act (WMO) and other guidelines, regulations and Acts. The study was registered at ClinicalTrials.gov before recruitment of the first participant (NCT03413254, first posted in January 2018).

Communication about patients will occur with the assigned study number. The full name and birth date of the patient will only be recorded on the informed consent form. In order to maintain confidentiality during and for fifteen years after completion of the study, all study-related information will be stored using the assigned study number in a secure and accessible place and manner. Digital files will be stored on password-protected computers in password-protected folders. Only the project leader and study coordinator have full access to the complete final dataset.

The AMC Medical Research BV has an insurance, which is in accordance with the legal requirements in the Netherlands (Article 7 WMO). This insurance provides cover for damage to research subjects through injury or death caused by the study. The insurance applies to the damage that becomes apparent during the study or within 4 years after.

### Dissemination

The COLOPEC 2 trial is designed within the research networks of DPOG and DCCG. The DPOG is a national multidisciplinary working group with the common goal of improving the treatment of peritoneal malignancies by carrying out multidisciplinary scientific research. Progress and final results of the study will be discussed during the regular meetings of both research groups, and updates will be available on the website. The results of the COLOPEC 2 trial will be presented at national and international congresses and submitted for publication to a peer-reviewed scientific journal.

## Discussion

### The use of second look surgery for detection of PM in pT4 colon cancer patients

There is no published trial on routine second look surgery in proven pT4 colon cancer patients. The COLOPEC 2 trial has some overlap with a currently recruiting Italian randomized trial investigating the role of second look surgery six months postoperatively in mucinous CRC (NCT01628211). All other ongoing trials are addressing the role of simultaneous prophylactic HIPEC in *clinical* T4 stage colon cancer: the PROMENADE trial (NCT02974556), and an almost similar Spanish multicentre study (NCT02614534). The inclusion criteria of these trials are essentially different from the COLOPEC 2, because the COLOPEC 2 is based on *pathological* T4 stage, thereby also including pT4a tumours that are often missed based on clinical staging. Furthermore, the COLOPEC 2 will investigate the impact of just DLS and only CRS/HIPEC if PM are found. Finally the role of third look laparoscopy has never been investigated. In the majority of other previously published and ongoing clinical trials concerning the use of second look surgery for early detection of PM, the patient group consists of patients with resected minimal synchronous macroscopic PM, synchronous ovarian metastases or perforation of the primary tumour. In our opinion, local peritoneal nodules that were resected at the time of primary surgery and resected ovarian metastases should be considered as already proven PM instead of risk factors for developing PM. According to the Dutch guideline, these patients would not have been treated with systemic therapy first, but would routinely be treated by upfront CRS/HIPEC immediately after the diagnosis of local peritoneal nodules or ovarian metastases.

Selection of patients at high risk of developing PM, but who were never diagnosed with PM, is another clinical scenario. Several studies have identified an advanced tumour stage as an independent risk factor for the development of PM [30–32, 39]. Except for a Chinese trial (NCT02179489) including both patients with proven PM as well as patients at high risk of developing PM (i.e. pT4 colon cancer and/or tumour perforation), studies regarding the impact of second look surgery in pT4 patients are lacking. Therefore, we think the COLOPEC 2 trial has a unique trial design, which essentially differs from previous and ongoing trials in this field.

### Abdominal CT and second look DLS as standard components of follow-up

CT imaging of the abdomen at 6 and 18 months can be considered a standard diagnostic tool according to the Dutch guideline, in which CT is recommended instead of ultrasound of the liver in patients at high risk of developing recurrence at 6 months interval in the first 2 years and yearly until 5 years postoperatively [37]. The second look DLS is currently not included in the Dutch guideline. However, second look surgery is increasingly applied in patients at high risk of developing PM in- and out-side trial setting. The preliminary findings of the COLOPEC trial and the increasing body of literature on risk of developing PM justify the systematic performance of second look DLS in this study. The results of this study will contribute to the evidence on second look DLS as an essential component in the follow-up of patients with pT4 colon cancer.

### Rationale for design; timing of DLS

According to the Dutch guideline, adjuvant systemic chemotherapy is indicated after curative intent resection of all pT4 cancers, both stage II and stage III, and should start preferably within 8 weeks and seems not beneficial if started after more than 3 months postoperatively [37]. Standard duration of adjuvant chemotherapy is 3 to 6 months. A substantial number of patients with pT4 colon cancer will not receive adjuvant systemic chemotherapy, because they experience complications or are unfit to start adjuvant systemic therapy within 12 weeks postoperatively [34]. Second look DLS in the 2 months postoperative period as performed in the COLOPEC trial has the disadvantage of delaying systemic chemotherapy. In the COLOPEC 2 trial, this delay cannot be justified, since only 10% of patients will benefit from the second look DLS, while the remaining 90% of patients might harm from the delay. Furthermore, such an early DLS is not possible in patients with abdominal complications (i.e. anastomotic leakage, abscess, fascial dehiscence), and might also be difficult after uncomplicated resections because of fresh adhesions, for example after open multivisceral resection. Finally, one might hypothesize that patients with rapid disease progression even before start of chemotherapy or during adjuvant treatment are probably beyond any curative intent treatment and will therefore not benefit from early detection. Therefore, DLS between 6 and 10 months postoperatively, depending on the delivery and duration of adjuvant chemotherapy, is regarded the most optimal timing to detect early development of metachronous PM at a curable stage. With CT-imaging, approximately 90% of PM are found within three years after primary resection [40]. It is expected that these PM can be detected at an earlier stage using DLS. Based on this assumption, the timing of the third look DLS between 18 and 20 months postoperative was chosen.

Duration of adjuvant chemotherapy for stage III colon cancer is currently subject of debate. A collaboration of six trials including 12.834 patients with stage III colon cancer showed an absolute difference of only 0.9% in 3-year disease-free survival after 3 months adjuvant chemotherapy compared to 6 months, while reducing the risk of neurotoxicity and other adverse events [41]. Changing recommendations in duration of adjuvant chemotherapy in the Dutch colorectal cancer guideline might positively influence the timing of second look DLS in the COLOPEC 2 trial, because this will allow for an increasing number of patients to be evaluated at 6 months postoperatively, irrespective of receiving adjuvant chemotherapy.

### Costs

There may be concerns about the additional costs related to the DLS. However, it is expected that these additional costs will weigh against gained life years and reduced costs of patients in whom PM would have been detected at a clinically apparent stage if no third look DLS was performed. These reduced costs will be the result of less postoperative morbidity and less need of palliative interventions, if PM are more often detected at a clinically occult stage.

## Additional file

Additional file 1: Recommended items to address in a clinical trial protocol and related documents. (DOCX 49 kb)

## Abbreviations

AE: Adverse event
CEA: Carcinoembryonic antigen
CRC: Colorectal cancer
CRS: Cytoreductive surgery
CT: Computed tomography
DLS: Diagnostic laparoscopy
DPOG: Dutch Peritoneal Oncology Group
DSMB: Data and Safety Monitoring Board
eCRF: Electronic case record form
GCP: Good clinical practice
HIPEC: Hyperthermic intraperitoneal chemotherapy
IKNL: Netherlands Comprehensive Cancer Organization (in Dutch: Integraal Kankercentrum Nederland)
IRB: Institutional Review Board
ISO: International Organization for Standardization
PCI: Peritoneal cancer index
PM: Peritoneal metastases
SAE: Serious adverse event
SPIRIT: Standard Protocol Items: Recommendations for Interventional Trials
WMO: Medical Research Involving Human Subjects Act (in Dutch: Wet Medisch-wetenschappelijk Onderzoek met mensen)
